# The Relationship Between Plant‐Based Diet Index and Quality of Life, Serum Levels of Pentraxin‐3, and Handgrip Strength in Patients on Maintenance Hemodialysis

**DOI:** 10.1002/fsn3.71535

**Published:** 2026-02-26

**Authors:** Sara Ghaedi, Sahar Foshati, Siavash Babajafari, Fatemeh Navab, Alieh Gholaminejad, Mojgan Mortazavi, Mohammad Hossein Rouhani

**Affiliations:** ^1^ Student Research Committee Shiraz University of Medical Sciences Shiraz Iran; ^2^ Department of Clinical Nutrition, School of Nutrition and Food Sciences Shiraz University of Medical Sciences Shiraz Iran; ^3^ Nutrition Research Center Shiraz University of Medical Sciences Shiraz Iran; ^4^ Nutrition and Food Security Research Center, Department of Community Nutrition, School of Nutrition and Food Science Isfahan University of Medical Sciences Isfahan Iran; ^5^ Regenerative Medicine Research Center, Faculty of Medicine Isfahan University of Medical Sciences Isfahan Iran; ^6^ Kidney Diseases Research Center Isfahan University of Medical Sciences Isfahan Iran

**Keywords:** hemodialysis, muscle strength, pentraxin‐3, plant‐based diet, quality of life

## Abstract

Hemodialysis (HD) patients often suffer from poor quality of life (QoL), malnutrition, and chronic inflammation. This study aimed to examine how plant‐based diet index (PDI) affects serum pentraxin‐3 (PTX3) levels (an inflammation marker), handgrip strength (HGS), and QoL in these patients. This cross‐sectional study was conducted on 321 HD patients from six medical centers in Isfahan, Iran. PDI was assessed using a 168‐item food frequency questionnaire. PTX3 levels were measured through the enzyme‐linked immunosorbent assay (ELISA). HGS was assessed using a dynamometer. QoL was evaluated using the Kidney Disease Quality of Life‐Short Form (KDQOL‐SF) questionnaire. Statistical data analysis included binary logistic regression and was adjusted for various confounders. HD Patients in the highest PDI tertile had significantly lower risk of high PTX3 levels (OR: 0.38, CI, 0.16–0.90) as well as lower risk of low HGS (OR: 0.43, CI, 0.19–0.94) compared to the lowest PDI tertile. Males with higher PDI scores also had significantly lower risk of low QoL (OR: 0.42, CI, 0.18–0.98), though no significant association was observed in females. Adherence to a plant‐based diet was associated with reduced inflammation, improved physical strength, and better QoL, particularly in male HD patients. A plant‐based diet may offer health benefits for HD patients, but further studies are needed to establish causality.

## Graphical abstract

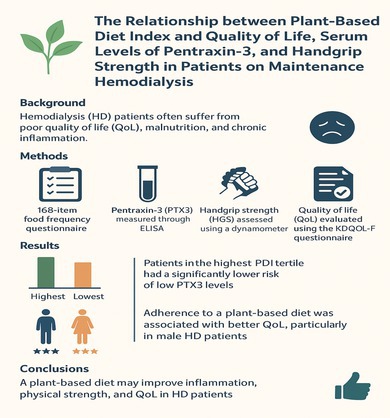

**Abbreviations**
BMI: body mass index
CKD: chronic kidney disease
CRP: c‐reactive protein
DAL: dietary acid load
ELISA: enzyme‐linked immunosorbent assay
ESRD: end stage renal disease
FFQ: food frequency questionnaire
HD: hemodialysis
HGS: handgrip strength
IL‐6: interleukin‐6
Kt/V: urea clearance × dialysis time/urea distribution volume
PDI: plant‐based diet index
PTX3: pentraxin‐3
QoL: quality of life
URR: urea reduction ratio

## 1 Background

Chronic kidney disease (CKD) is a persisting condition of renal damage that has affected more than 10% of the world's population, encompassing a staggering population of over 800 million individuals. Some special populations such as the elderly, women, individuals with diabetes and hypertension, and ethnic minorities are more vulnerable to this condition (Kovesdy 2022). Annually, CKD and its associated complications impose a substantial economic burden on societies and stand as a significant contributor to mortality, ranking among the leading causes of death worldwide (Dehvan et al. 2018).

Hemodialysis (HD) is a therapeutic approach for people in the advanced stages of CKD, that is. end stage renal disease (ESRD), to remove extra fluid and wastes from the blood. Approximately 2 million individuals undergo HD worldwide (Inker et al. 2014; Msaad et al. 2019). Despite technological advancements in medicine, ESRD patients undergoing HD have higher hospitalization rates, lower quality of life (QoL) scores—defined as a patient's perceived well‐being across physical, emotional, and social domains—increased bacterial and viral infection rates, and elevated mortality risk compared to the majority of non‐dialysis populations (Chandrashekar et al. 2014). The mechanisms underlying these adverse outcomes are multifactorial, with malnutrition and inflammation playing pivotal and intertwined roles that contribute to poor QoL and worse prognosis (Liakopoulos et al. 2017; Young et al. 2011). Moreover, the majority of HD patients are afflicted by protein‐energy malnutrition (Dukkipati and Kopple 2009). (Keane et al. 2016) reported that body composition of ESRD patients changed significantly within 2 years of commencing HD, indicating a reduction in fat‐free mass and an increase in fat mass. In addition, some studies showed that handgrip strength (HGS), an indicator of functional ability and nutritional status, was low in patients on HD (Hasheminejad et al. 2016; Tian et al. 2019). Moreover, serum levels of pentraxin‐3 (PTX3), a multimeric acute phase inflammatory glycoprotein, were reported to increase in HD patients and may predispose them to oxidative stress, malnutrition, cardiovascular diseases, and renal tissue fibrosis (Valente et al. 2019; Zhou et al. 2013). Together, these measures reflect a network of inflammation, nutrition, and function that shapes QoL in HD.

Plant‐based dietary patterns are increasingly recommended for CKD/ESRD patients to improve nutrient intake and metabolic health, though concerns about potassium‐rich foods and overall energy balance persist in HD cohorts (Joshi et al. 2021; Noori et al. 2010). Emerging evidence suggests that higher fruit and vegetable intake may help mitigate metabolic acidosis and may slow CKD progression, with potential benefits extending to inflammation and nutritional status (St‐Jules et al. 2016). Yet, knowledge gaps remain, particularly regarding how plant‐based dietary patterns relate to inflammatory markers (e.g., PTX3), functional measures (HGS), and QoL in HD patients, and whether these associations vary by sex or other demographic factors (Xie et al. 2015; Carrero et al. 2020). Therefore, the present study aimed to investigate the association between plant‐based diet index (PDI) and serum concentrations of PTX3, HGS, and overall QoL in patients on maintenance HD. We also explore potential sex differences in these associations to inform targeted dietary strategies in this high‐risk population.

## 2 Methods

### 2.1 Study Design and Participants

This cross‐sectional study was carried out between April 2022 and September 2023 across six main HD centers in Isfahan, Iran, including Farabi, Khorshid, Shariati, Amin, Hojjatieh, and Zahraye Marzieh Hospitals. Eligible participants were adults aged 18 years or older who had been receiving maintenance HD for at least 90 days and were both able and willing to take part in the study. Individuals were excluded if they reported implausible daily energy intakes (< 800 kcal/day or > 4200 kcal/day) (Banna et al. 2017), received enteral or parenteral nutrition, had a known history of cancer, myocardial infarction, or severe hepatic disease, were active smokers, or had been recently hospitalized or infected (within the previous month).

### 2.2 Sample Size Estimation

The sample size was estimated using the formula (*N* = [(Z_1 − α/2_)^2^ × SD^2^]/d^2^) (Charan and Biswas 2013), with PTX3 concentration as the key variable. The primary variable employed to compute the sample size was PTX3. Based on prior Iranian research, the SD for PTX3 was 2.5 ng/mL (Gotch and Sargent 1985). With a significance level of 0.05 and precision (d) of 0.33 ng/mL, a minimum of 221 participants was required. To increase statistical power and account for potential exclusions, a total of 321 patients were ultimately recruited.

### 2.3 Research Ethics and Participant Authorization

All participants provided written informed consent prior to the study. The research was performed in accordance with the ethical principles of the Declaration of Helsinki and approved by the Ethics Committee of Shiraz University of Medical Sciences (Approval Code: IR.SUMS.REC.1403.119).

### 2.4 Demographic and Clinical Data

Information regarding age, marital status, and employment was obtained through interviewer‐administered questionnaires. Additional medical data—such as the primary cause of kidney failure, duration and frequency of HD sessions, concomitant illnesses, and prescribed medications—were retrieved from medical records.

### 2.5 Assessment of Dialysis Adequacy

Dialysis adequacy was assessed using both the urea reduction ratio (URR) and the Kt/V index. URR was determined by the equation: URR = {(Blood urea_predialysis_ − Blood urea_postdialysis_)/Blood urea_predialysis_} × 100. Kt/V was calculated by multiplying urea clearance (K) by dialysis time (t) and dividing by the urea distribution volume (V), as recommended by standard clinical guidelines (Maduell et al. 1998).

### 2.6 Anthropometric Measurements

Body weight was recorded immediately after the dialysis session when patients were in a euvolemic state and wearing light clothing (Sinha and Agarwal 2017), using a calibrated Seca digital scale (Seca Co., Hamburg, Germany) with 0.1 kg precision. Height was measured to the nearest 0.1 cm without shoes using a stadiometer. Body mass index (BMI) was computed as weight (kg) divided by height squared (m^2^).

### 2.7 Dietary Intake Assessment

Participants' habitual dietary intake over the preceding year was evaluated using a semi‐quantitative 168‐item food frequency questionnaire (FFQ), previously validated for use in Iranian populations (Mirmiran et al. 2010). Trained dietitians conducted face‐to‐face interviews to record the average frequency of food intake on a daily, weekly, or monthly basis, using standardized portion sizes. Reported frequencies were converted into grams per day using common Iranian household measures (Ghafarpour et al. 1999). Macronutrient and micronutrient intakes were calculated using Nutritionist IV software (version 3.5.2, First Databank, Hearst Corp., USA).

### 2.8 PDI Calculation

PDI was calculated following the method first applied in the PREDIMED study (Martínez‐González et al. 2014), with minor adjustments to accommodate the local food database. Based on FFQ data, food items were categorized into healthy plant foods (fruits, vegetables, whole grains, legumes, nuts, seeds, tea/coffee, and plant oils), less‐healthy plant foods (refined grains, sweets, sugar‐sweetened beverages, and fried snacks), and animal‐based foods (meat, poultry, fish, eggs, dairy products, and animal fats). Participants in the highest quintile of healthy or less‐healthy plant food consumption received a score of 5, and those in the lowest received 1; the scoring was reversed for animal foods. The PDI was computed as the sum of all food‐group scores, with higher totals reflecting greater adherence to a plant‐based dietary pattern. The scoring criteria for PDI are detailed in Table S1.

### 2.9 Biochemical Assessment

Venous blood samples (5 mL) were collected from each participant after a 12‐h overnight fast and centrifuged at 2000 rpm for 10 min at 4°C to obtain serum. PTX3 concentrations were quantified via a commercially available ELISA kit (ZellBio GmbH, Lonsee, Germany), following the manufacturer's protocol. Inter‐ and intra‐assay coefficients of variation were < 10% and < 12%, respectively, confirming acceptable analytical precision.

### 2.10 HGS Evaluation

HGS was determined using a calibrated dynamometer according to the Southampton protocol (Roberts et al. 2011). According to the protocol and KDOQI guideline (Inker et al. 2014), measurements were taken on the non‐fistula hand before the dialysis session to avoid vascular complications. Each participant performed three maximal attempts with short rest intervals; the mean of the three readings was recorded for analysis.

### 2.11 QoL Assessment

QoL was measured using version 1.3 of the Kidney Disease Quality of Life‐Short Form (KDQOL‐SF) questionnaire. This instrument includes multiple CKD‐specific domains—such as symptom burden, sleep quality, cognitive and sexual function, social support, and patient satisfaction—as well as general health subscales from the SF‐36. Scores for each domain range from 0 to 100, with higher values indicating better‐perceived QoL. It is worth noting that the Persian version has previously demonstrated acceptable validity and reliability in Iranian HD populations (Pakpour et al. 2011).

### 2.12 Statistical Analysis

Statistical analyses were conducted using SPSS software version 19 (IBM, Chicago, IL), with *p* < 0.05 considered statistically significant. The Kolmogorov–Smirnov test was utilized to assess the normality of quantitative variables. Continuous variables were reported as mean ± standard deviation (SD) or standard error (SE), while qualitative variables were presented as frequency (percentage). Participants were divided into tertiles based on their scores in PDI. To compare quantitative and categorical variables among these tertiles, one‐way analysis of variance (ANOVA) and the chi‐squared test were employed, respectively. Dietary intakes adjusted for age, sex, and energy intake were analyzed across the PDI tertiles using analysis of covariance (ANCOVA). For variables with a significant ANCOVA result, Tukey post hoc tests were used to compare tertiles pairwise (T1 vs. T2, T1 vs. T3, T2 vs. T3). QoL, PTX3, and HGS were dichotomized based on sex‐specific median values (high/low) to facilitate interpretation and model convergence due to non‐normal distributions. Binary logistic regression was used to determine the relationship between the tertiles of PDI and low QoL, high PTX3 levels, and low HGS, with odds ratio (OR) and 95% confidence interval (CI) calculated for both crude and adjusted models. The first model adjusted for sex and age; the second model included additional adjustments for job, marital status, and URR; the final model further adjusted for energy intake. In all models, the first tertile (T1) of PDI served as the reference category. The trend of OR across increasing tertiles was analyzed by treating PDI tertiles as an ordinal variable.

## 3 Results

### 3.1 General Characteristics

The participants shared similar demographic and baseline characteristics across the tertiles of PDI, with no significant differences detected (Table 1). This overall balance among groups strengthens the internal validity of the study results and enhances confidence that differences identified in subsequent analyses primarily reflect variations in dietary patterns rather than underlying participant characteristics.

**TABLE 1:** General characteristics of study participants across tertiles of PDI.

	Tertiles of PDI			
	T1 (*n* = 106) range < 51	T2 (*n* = 112) range 51–57	T3 (*n* = 103) range > 57	*p*
Sex				0.84
Male	77 (72.6)	82 (73.2)	72 (69.9)	
Female	29 (27.4)	30 (26.8)	31 (30.1)	
Age (years)	61.04 ± 15.32	56.81 ± 15.97	57.41 ± 14.25	0.09
BMI				0.46
Underweight	6 (7.1)	6 (6.3)	9 (11.1)	
Normal weight	43 (51.2)	52 (54.2)	32 (39.5)	
Overweight	25 (29.8)	27 (28.1)	32 (39.55)	
Obese	10 (11.9)	11 (11.5)	8 (9.9)	
Marital status				0.31
Single	12 (12.4)	19 (19.8)	12 (13.6)	
Married	85 (87.6)	77 (80.2)	76 (86.4)	
Job status				0.18
Housewife	27 (25.5)	25 (22.3)	21 (20.4)	
Employed	8 (7.5)	8 (7.1)	10 (9.7)	
Freelance	20 (18.9)	20 (17.9)	14 (13.6)	
Unemployed	11 (10.4)	29 (25.9)	23 (22.3)	
Retired	40 (37.7)	30 (26.8)	35 (34.0)	
Cause of renal failure				0.49
Diabetes	52 (49.1)	47 (42.3)	40 (38.8)	
Hypertension	31 (29.2)	33 (29.7)	29 (28.2)	
AKI	1 (0.9)	4 (3.6)	2 (1.9)	
Other	22 (20.8)	27 (24.3)	32 (31.1)	
Dialysis vintage (months)	44.03 ± 38.68	53.08 ± 53.61	45.44 ± 40.03	0.28
Dialysis sessions per month	11.06 ± 2.032	11.03 ± 2.027	10.41 ± 2.90	0.10
Kt/V	1.32 ± 0.23	1.36 ± 0.21	1.32 ± 0.24	0.34
URR	0.72 ± 0.16	0.71 ± 0.12	0.70 ± 0.14	0.55

*Note:* Values are mean ± SD for quantitative variables and number (percentage) for qualitative ones. One‐way ANOVA test and *χ*2 test for quantitative and qualitative variables, respectively.

Abbreviations: AKI, acute kidney injury; BMI, body mass index; Kt/V, clearance of urea multiplied by dialysis duration and normalized for urea distribution volume; PDI, plant‐based diet index; URR, urea reduction ratio.

### 3.2 Dietary Intakes

Dietary intake analysis revealed distinct nutritional variations across PDI tertiles (Table 2). In particular, participants in the highest tertile (T3) consumed significantly more total energy than those in T2 and T1. Carbohydrate intake also increased progressively across tertiles (T3 > T2 > T1). Similarly, magnesium, thiamine, folate, and sodium intakes were highest in T3, intermediate in T2, and lowest in T1, with all pairwise comparisons reaching statistical significance. In contrast, total fat, saturated fat, monounsaturated fat, polyunsaturated fat, cholesterol, and vitamin B12 intakes were significantly lower in T3 compared with both T2 and T1, with T1 and T2 not differing from each other for these nutrients. Nevertheless, no significant differences were observed for the remaining dietary components.

**TABLE 2:** Dietary intakes (energy and macro/micronutrients) of study participants across tertiles of PDI.

	Tertiles of PDI			
	T1 (*n* = 106) range < 51	T2 (*n* = 112) range 51–57	T3 (*n* = 103) range > 57	*p*
Energy (kcal/d)	1355.04 ± 72.07^a^	1747.79 ± 70.22^b^	2400.87 ± 73.16^c^	< 0.001
Protein (g/d)	68.17 ± 1.89	68.61 ± 1.73	62.78 ± 1.97	0.072
Carbohydrate (g/d)	247.95 ± 5.33^a^	270.66 ± 4.88^b^	302.13 ± 5.55^c^	< 0.001
Fat (g/d)	68.25 ± 2.66^a^	58.61 ± 2.43^a^	47.94 ± 2.77^b^	< 0.001
Cholesterol (mg/d)	238.22 ± 9.98^a^	215.24 ± 9.14^a^	154.51 ± 10.40^b^	< 0.001
SFA (g/d)	21.24 ± 0.71^a^	19.73 ± 0.65^a^	15.67 ± 0.73^b^	< 0.001
MUFA (g/d)	24.37 ± 1.19^a^	20.22 ± 1.09^a^	15.74 ± 1.24^b^	< 0.001
PUFA (g/d)	14.92 ± 1.13^a^	11.01 ± 1.04^a^	9.37 ± 1.18^b^	0.004
Vitamin C (mg/d)	138.24 ± 7.99	149.42 ± 7.32	147.82 ± 8.32	0.564
Vitamin A (RE/d)	504.90 ± 27.68	525.57 ± 25.36	516.72 ± 28.84	0.857
Thiamin (mg/d)	1.36 ± 0.04^a^	1.41 ± 0.03^b^	1.52 ± 0.04^c^	0.035
Riboflavin (mg/d)	1.67 ± 0.05	1.70 ± 0.05	1.54 ± 0.06	0.130
Niacin (mg/d)	18.27 ± 0.59	18.63 ± 0.54	17.77 ± 0.62	0.588
Vitamin B6 (mg/d)	1.68 ± 0.04	1.77 ± 0.04	1.71 ± 0.04	0.344
Vitamin E (mg/d)	12.78 ± 0.97	10.82 ± 0.88	9.47 ± 1.01	0.082
Folate (mcg/d)	405.81 ± 13.07^a^	442.47 ± 11.97^b^	495.67 ± 13.62^c^	< 0.001
Vitamin B12 (mcg/d)	3.36 ± 0.16^a^	3.23 ± 0.15^a^	2.37 ± 0.17^b^	< 0.001
Magnesium (mg/d)	319.31 ± 9.58^a^	350.56 ± 8.77^b^	369.19 ± 9.98^c^	0.003
Zinc (mg/d)	10.13 ± 0.31	10.61 ± 0.29	10.29 ± 0.33	0.512
Total fiber (g/d)	79.81 ± 2.92	85.56 ± 2.68	89.05 ± 3.05	0.115
Sodium (mg/d)	2917.18 ± 146.02^a^	3515.47 ± 133.76^b^	3569.22 ± 152.13^c^	0.004
Potassium (mg/d)	3518.69 ± 126.97	3824.48 ± 116.30	3784.89 ± 132.27	0.182

*Note:* Values are mean ± SE. Energy intake was adjusted for age and sex; all other values were adjusted for age, sex, and energy intake (by the use of ANCOVA). For variables with a significant ANCOVA result, Tukey post hoc tests were used to compare tertiles pairwise (T1 vs. T2, T1 vs. T3, T2 vs. T3). Superscript letters (a, b, c) indicate significant differences at *p* < 0.05. Means sharing the same letter are not significantly different.

Abbreviations: MUFA, monounsaturated fatty acids; PUFA, polyunsaturated fatty acids; PDI, plant‐based diet index; SFA, saturated fatty acids.

### 3.3 QoL

Regarding QoL, individuals in the highest PDI tertile demonstrated a substantially lower likelihood of low QoL compared with those in the lowest tertile (Table 3). This association remained significant after adjusting for age, sex, job, marital status, and URR (Models 1 and 2), but became non‐significant after further adjustment for energy consumption (Model 3). Stratified analysis showed that this association was more pronounced among men, even in Model 3, where those in T3 had a 58% lower risk of low QoL compared to T1 (OR = 0.42, 95% CI, = 0.18–0.98), while no significant association was found among women.

**TABLE 3:** The association between quality of life and tertiles of PDI stratified by sex categories.

	Tertiles of PDI			
	T1 (*n* = 106) range < 51	T2 (*n* = 112) range 51–57	T3 (*n* = 103) range > 57	*p*‐trend
**Total population**				
Crude	1 (Ref.)	0.76 (0.44, 1.30)	0.46 (0.27, 0.81)	0.007
Model 1	1 (Ref.)	0.82 (0.47, 1.42)	0.48 (0.27, 0.85)	0.012
Model 2	1 (Ref.)	0.85 (0.47, 1.53)	0.49 (0.26, 0.90)	0.025
Model 3	1 (Ref.)	1.02 (0.56, 1.85)	0.55 (0.26, 1.13)	0.169
**Male**				
Crude	1 (Ref.)	0.68 (0.36, 1.27)	0.35 (0.18, 0.68)	0.009
Model 1	1 (Ref.)	0.73 (0.39, 1.39)	0.36 (0.18, 0.71)	0.012
Model 2	1 (Ref.)	0.80 (0.40, 1.58)	0.36 (0.17, 0.76)	0.024
Model 3	1 (Ref.)	1.00 (0.48, 2.07)	0.42 (0.18, 0.98)	0.075
**Female**				
Crude	1 (Ref.)	1.05 (0.36, 3.03)	0.84 (0.30, 2.38)	0.907
Model 1	1 (Ref.)	1.13 (0.38, 3.30)	0.98 (0.33, 2.83)	0.960
Model 2	1 (Ref.)	1.01 (0.33, 3.17)	1.00 (0.31, 3.13)	0.999
Model 3	1 (Ref.)	1.05 (0.35, 3.10)	1.24 (0.23, 6.60)	0.966

*Note:* Values are odds ratio (95% confidence interval). Binary logistic regression was used for crude and adjusted models. Model 1: Adjusted for age and sex. Model 2: Additionally, adjusted for job, marital status, and URR. Model 3: Additionally adjusted for energy intake. *p*‐values were obtained by the use of tertiles of PDI as an ordinal variable in the model.

Abbreviation: PDI, plant‐based diet index.

### 3.4 PTX‐3

An interesting pattern was observed in the association between PDI and PTX3 levels (Table 4). Although no significant differences were evident in the crude and minimally adjusted models, further adjustment for confounders revealed a markedly lower risk of elevated PTX3 levels among participants in the highest PDI tertile (Model 2: OR = 0.46, 95% CI, = 0.22–0.94; Model 3: OR = 0.38, 95% CI, = 0.16–0.90). Sex‐specific analyses again indicated that this inverse association was significant only among men, who showed a 68% lower risk of high PTX3 levels in T3 compared with T1 (OR = 0.32, 95% CI, = 0.11–0.88).

**TABLE 4:** The association between pentraxin‐3 levels and tertiles of PDI stratified by sex categories.

	Tertiles of PDI			
	T1 (*n* = 106) range < 51	T2 (*n* = 112) range 51–57	T3 (*n* = 103) range > 57	*p*‐trend
**Total population**				
Crude	1 (Ref.)	0.52 (0.28, 0.99)	0.55 (0.28, 1.06)	0.093
Model 1	1 (Ref.)	0.52 (0.27, 0.98)	0.54 (0.28, 1.05)	0.089
Model 2	1 (Ref.)	0.49 (0.25, 0.98)	0.46 (0.22, 0.94)	0.056
Model 3	1 (Ref.)	0.42 (0.21, 0.84)	0.38 (0.16, 0.90)	0.025
**Male**				
Crude	1 (Ref.)	0.43 (0.20, 0.91)	0.44 (0.20, 0.96)	0.047
Model 1	1 (Ref.)	0.43 (0.20, 0.92)	0.44 (0.20, 0.97)	0.051
Model 2	1 (Ref.)	0.39 (0.17, 0.91)	0.33 (0.14, 0.80)	0.026
Model 3	1 (Ref.)	0.31 (0.13, 0.75)	0.32 (0.11, 0.88)	0.020
**Female**				
Crude	1 (Ref.)	0.90 (0.26, 3.07)	0.99 (0.28, 3.43)	0.982
Model 1	1 (Ref.)	0.88 (0.24, 3.04)	0.96 (0.27, 3.35)	0.981
Model 2	1 (Ref.)	0.77 (0.22, 2.74)	0.93 (0.24, 3.53)	0.920
Model 3	1 (Ref.)	0.58 (0.17, 1.95)	0.36 (0.05, 2.42)	0.524

*Note:* Values are odds ratio (95% confidence interval). Binary logistic regression was used for crude and adjusted models. Model 1: Adjusted for age and sex. Model 2: Additionally, adjusted for job, marital status, and URR. Model 3: Additionally adjusted for energy intake. *p*‐values were obtained by the use of tertiles of PDI as an ordinal variable in the model.

Abbreviation: PDI, plant‐based diet index.

### 3.5 HGS

Finally, HGS analyses revealed that participants in the highest PDI tertile had a 57% lower risk of low HGS compared with those in the lowest tertile in the fully adjusted model, that is Model 3 (OR = 0.43, 95% CI, = 0.19–0.94) (Table 5). Although this relationship was not significant in earlier adjusted models or when stratified by sex, the overall trend suggests a potential beneficial effect of higher adherence to a plant‐based diet on muscle strength among HD patients.

**TABLE 5:** The association between handgrip strength and tertiles of PDI stratified by sex categories.

	Tertiles of PDI			
	T1 (*n* = 106) range < 51	T2 (*n* = 112) range 51–57	T3 (*n* = 103) range > 57	*p*‐trend
**Total population**				
Crude	1 (Ref.)	0.69 (0.40, 1.21)	0.55 (0.30, 0.98)	0.123
Model 1	1 (Ref.)	0.81 (0.45, 1.46)	0.62 (0.33, 1.13)	0.297
Model 2	1 (Ref.)	0.74 (0.40, 1.40)	0.51 (0.26, 1.00)	0.147
Model 3	1 (Ref.)	0.70 (0.37, 1.33)	0.43 (0.19, 0.94)	0.112
**Male**				
Crude	1 (Ref.)	0.82 (0.43, 1.56)	0.58 (0.29, 1.15)	0.293
Model 1	1 (Ref.)	1.01 (0.51, 2.01)	0.65 (0.31, 1.35)	0.412
Model 2	1 (Ref.)	0.89 (0.40, 1.94)	0.51 (0.22, 1.17)	0.246
Model 3	1 (Ref.)	0.83 (0.36, 1.91)	0.38 (0.15, 1.00)	0.120
**Female**				
Crude	1 (Ref.)	0.43 (0.14, 1.29)	0.47 (0.15, 1.39)	0.257
Model 1	1 (Ref.)	0.46 (0.15, 1.44)	0.51 (0.17, 1.57)	0.355
Model 2	1 (Ref.)	0.46 (0.14, 1.46)	0.51 (0.15, 1.68)	0.376
Model 3	1 (Ref.)	0.41 (0.13, 1.25)	0.61 (0.11, 3.18)	0.291

*Note:* Values are odds ratio (95% confidence interval). Binary logistic regression was used for crude and adjusted models. Model 1: Adjusted for age and sex. Model 2: Additionally, adjusted for job, marital status, and URR. Model 3: Additionally adjusted for energy intake. *p*‐values were obtained by the use of tertiles of PDI as an ordinal variable in the model.

Abbreviation: PDI, plant‐based diet index.

## 4 Discussion

This cross‐sectional study sheds light on the associations between adherence to a plant‐based diet, as measured by the PDI, and various health outcomes within the total population of HD patients and separately in male and female populations. The results indicate that males who adhered more closely to a plant‐based diet exhibited a higher QoL (an indicator of both physical and mental health) compared to those with lower adherence. In addition, the study found that both in the total population and specifically among males, participants with higher PDI scores had lower levels of PTX3, a biomarker associated with inflammation and cardiovascular risk. Moreover, in the total population, those with higher PDI scores also demonstrated greater HGS, which is an indicator of better physical function and muscle strength and can contribute to improved mental health through greater independence and confidence. These findings highlight the potential of a plant‐based diet to positively influence both physical and mental health.

### 4.1 QoL

Previous studies have consistently shown that patients undergoing HD often experience a reduced QoL due to both physical and mental challenges associated with their condition (Gerasimoula et al. 2015). No studies have yet investigated the impact of the PDI on QoL in HD patients, but previous research has demonstrated that plant protein intake can favorably influence kidney function and reduce complications in these patients (Dupuis et al. 2021; He et al. 2021). In our study, greater adherence to the PDI was associated with an improved QoL in the male HD population. Mechanistically, sex‐differences in muscle mass, hormone profiles (e.g., androgens, estrogens), and inflammatory regulation may make men more responsive to PDI in ways that reduce systemic inflammation and preserve muscle strength, thereby improving QoL. In women, psychosocial factors and potential differences in nutrient requirements or metabolism may attenuate these effects. In addition, unmeasured confounding variables and unequal sample sizes by sex may further exaggerate or obscure the true mechanisms (Marchese et al. 2025).

A potential explanation for the positive effect between PDI and QoL is that consuming more plant foods can lower the dietary acid load (DAL), and low DAL has been reported to delay renal dysfunction. DAL reflects the net acid‐forming potential of the diet. A high DAL, typical in animal‐protein‐rich diets, may exacerbate metabolic acidosis in HD patients. Metabolic acidosis in ESRD patients on HD can worsen insulin resistance, promote bone demineralization, accelerate muscle protein catabolism, and increase the risk of inflammation and malnutrition. In contrast, plant‐based diets lower DAL and may improve physical and mental outcomes (Mirmiran et al. 2016). Daneshzad et al.'s study (Daneshzad et al. 2020) has also indicated that a lower DAL is associated with a decreased risk of depression and sleep disorders. Therefore, plant foods may have a beneficial impact on mental health and QoL due to their low DAL. Furthermore, a diet rich in plant‐based foods may improve appetite and caloric intake, thereby lowering the risk of malnutrition and ultimately enhancing QoL (Joshi et al. 2019).

### 4.2 PTX‐3

The present study demonstrates that higher adherence to the PDI is associated with lower levels of PTX3, an inflammatory biomarker linked to cardiovascular disease and mortality, in both the overall population and the male subset of HD patients. Supporting evidence from a systematic review and meta‐analysis suggests that the consumption of animal proteins may be associated with elevated levels of CRP compared to the intake of other protein sources, such as plant‐based proteins, among dialysis participants (Aycart et al. 2021). Additionally, higher consumption of total red and processed meat has been associated with an increased risk of developing CKD, highlighting the potential benefits of plant‐based diets for this specific population (Mirmiran et al. 2020). The beneficial impact of a plant‐based diet on PTX3 levels in HD patients may be attributed to the high content of polyphenols, antioxidants, and fibers found in plant foods, which effectively reduce inflammatory markers (Xie et al. 2015; Rapa et al. 2019). Moreover, plant‐based foods contribute to a decrease in the production of uremic toxins by acting as prebiotics and positively influencing gut microbiota. This may help prevent inflammatory processes and mitigate the associated risk of cardiovascular disease, a leading cause of death, among HD patients (Montemurno et al. 2014; Rampton et al. 1984).

### 4.3 HGS

Our findings emphasize the significant association between higher PDI scores and greater HGS, which is essential for maintaining functional status and reducing the risk of malnutrition. Previous studies indicate that a carefully structured plant‐based diet can help maintain muscle strength (St‐Jules et al. 2019). This observation aligns with emerging research suggesting that plant‐based diets do not adversely affect the nutritional status of HD patients (González‐Ortiz et al. 2021). Moreover, it is well established that protein plays a critical role in regulating muscle metabolism, and insufficient protein intake can lead to sarcopenia, especially in people with kidney disease (Wang and Mitch 2014). However, the mixed results reported in other studies, such as those showing no change in HGS or fat‐free mass with increased plant protein intake, highlight the necessity for further research into the factors influencing these outcomes (Moorthi et al. 2014). In addition, the findings by (Susetyowati et al. 2023) support the notion that the primary concern for HD patients should be ensuring adequate energy and protein intake, irrespective of the protein source, to prevent malnutrition and its associated risks.

### 4.4 Strengths and Limitations

This study is the first to investigate the relationship between PDI and QoL, PTX3 levels, and HGS in HD patients. To obtain more accurate results, we analyzed this relationship separately for men and women while adjusting for the influence of key confounding factors. However, the study has several limitations, such as the lack of measurements for other inflammatory and oxidative biomarkers, which would have facilitated a more comprehensive examination of the associations. Furthermore, despite using a validated FFQ, there may be measurement errors and recall biases in assessing dietary intake. Moreover, although the cross‐sectional design limits causal inference, this study is valuable for uniquely integrating dietary patterns, biochemical markers (PTX3), and functional outcomes (HGS and QoL) in HD patients. It provides a strong rationale for future prospective studies to validate these associations.

## 5 Conclusions

In conclusion, this study suggests that adherence to a plant‐based diet is associated with improved QoL, reduced inflammation, and enhanced muscle strength in adult HD patients, particularly among males. These findings underscore the potential benefits of a plant‐based diet in promoting overall mental and physical health in this population.

## Author Contributions

**Sara Ghaedi:** writing – original draft, formal analysis, validation, visualization. **Sahar Foshati:** conceptualization, methodology, writing review and editing, formal analysis. **Siavash Babajafari:** supervision, project administration, funding acquisition. **Fatemeh Navab:** investigation, data curation. **Alieh Gholaminejad:** investigation, data curation. **Mojgan Mortazavi:** investigation, data curation. **Mohammad Hossein Rouhani:** resources, investigation, data curation.

## Funding

The present article was extracted from the MSc thesis written by Ms. Sara Ghaedi and was financially supported by Shiraz University of Medical Sciences and Isfahan University of Medical Sciences.

## Ethics Statement

Prior to the initiation of the study, all participants provided written informed consent. This study was conducted in accordance with the guidelines established in the Declaration of Helsinki, and all procedures involving human subjects were approved by the local ethics committee of Shiraz University of Medical Sciences (code: IR.SUMS.REC.1403.119).

## Conflicts of Interest

The authors declare no conflicts of interest.

## Supporting information


**Table S1:** Supporting Information.

## Data Availability

The data that underlie the results of this cross‐sectional study can be obtained by making a reasonable request to the corresponding authors.
