# Supplementary figures and images for: The Relationship Between Plant‐Based Diet Index and Quality of Life, Serum Levels of Pentraxin‐3, and Handgrip Strength in Patients on Maintenance Hemodialysis
Source: Food Sci Nutr. 2026 Feb 26;14(3):e71535. doi: 10.1002/fsn3.71535 (PMC12945706; doi:10.1002/fsn3.71535)

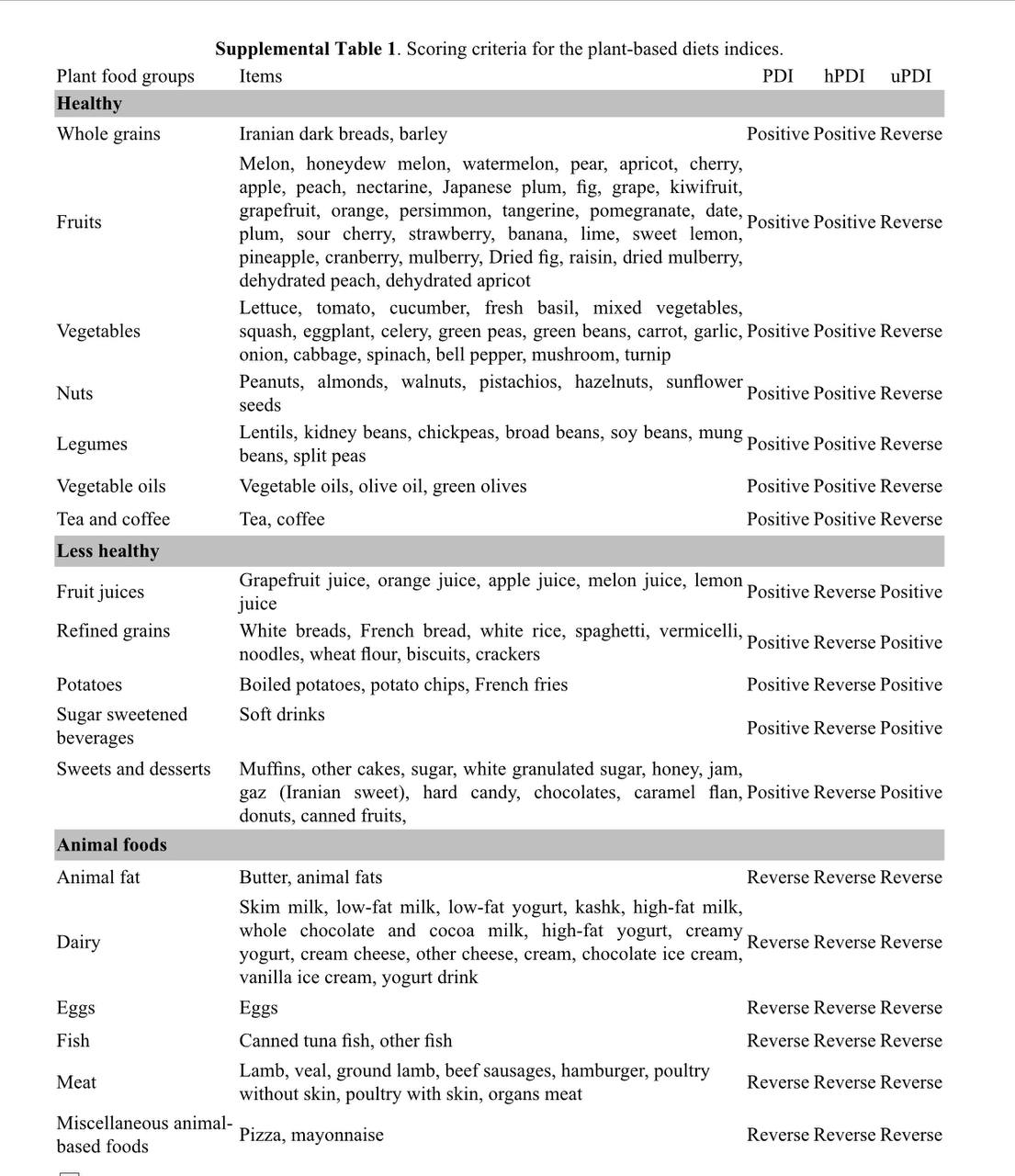

Supplement: Supplementary file 1 — Table S1: Supporting Information. [file FSN3-14-e71535-s001.doc]
